# Atypical Presentation of Acute Pericarditis Secondary to Allogeneic Hematopoietic Stem Cell Transplantation: A Case Report

**DOI:** 10.7759/cureus.44868

**Published:** 2023-09-07

**Authors:** Sourabh Goyal, Angela Khidhir, Matthew D Burwinkel

**Affiliations:** 1 Department of Medicine, University at Buffalo Jacobs School of Medicine and Biomedical Sciences, Buffalo, USA

**Keywords:** cancer therapy-related cardiac dysfunction, secondary acute myeloid leukemia, cardiotoxicity, atypical pericarditis, allogeneic hematopoietic stem cell transplantation

## Abstract

Cardiotoxicity linked with hematopoietic stem cell transplantation (HSCT) is a well-described phenomenon associated with an increased mortality risk; however, the majority of cardiac events present over 100 days following transfusion and are often attributed to graft-versus-host disease or pre-treatment conditioning by chemotherapy with or without radiation therapy. Here, we present the case of a 60-year-old female with a medical history of chronic lymphocytic leukemia complicated by a myelodysplastic syndrome that progressed to acute myeloid leukemia who developed chest pain immediately following an allogeneic HSCT. Electrocardiogram showed dynamic ST-depressions in leads V3-5 without evidence of reciprocal changes. Transthoracic echocardiography revealed pericardial effusion without signs of tamponade. The patient was thought to have acute pericarditis and was subsequently treated with high-dose intravenous methylprednisolone with a taper for two weeks. Her symptoms promptly subsided, and the pericardial effusion resolved on repeat echocardiography, which confirmed the diagnosis. Acute pericarditis is a rarely described complication of HSCT that is fatal if left untreated and prompts urgent management. This atypical case of acute pericarditis in the early post-transplant phase highlights the importance of cardiac stratification in patients with active malignancy undergoing treatment. It would suggest a potential benefit in closely monitoring high-risk individuals who have a history of coronary artery disease, smoking, or pericarditis in the pre-engraftment phase of transplantation.

## Introduction

Allogeneic hematopoietic stem cell transplantation (HSCT) is an increasingly used treatment of choice for a variety of hematological malignancies and other non-malignant hematological disorders due to its curative potential and improved overall survival [1,2]. The Health Resources and Services Administration reported 111,984 total HSCTs during 2016-2020, with approximately 22,000 transplants performed annually [2-4]. There have been significant advancements in HSCTs over recent decades to minimize risk profiles and improve outcomes, including the development of haploidentical transplants and non-myeloablative conditioning regimens [5]. However, despite these improvements, there are numerous adverse events associated with HSCTs that can impact morbidity, mortality, and quality of life [6].

Long-term adverse effects associated with HSCTs are established and can be life-threatening. HSCT-associated cardiotoxicity is estimated to account for 9.8% of cardiac events, including cardiac arrhythmias, acute heart failure, and hypertension with an increased one-year mortality risk [7,8]. Cardiovascular disease is a leading cause of excess deaths in five-year survivors at 11% [9]. However, pericarditis, specifically in the acute phase, is a lesser-known cardiotoxic complication with an observed 3.2% incidence and median time to onset of seven days post transfusion in a single-center retrospective study [10]. Here, we describe the unusual presentation of an adult patient affected by acute pericarditis on day 0 of transfusion.

## Case presentation

A 60-year-old female with a history of secondary myelodysplastic syndrome complicated by advanced acute myeloid leukemia presented for a scheduled allogeneic stem cell transplant. Her conditioning regimen consisted of weight-based dosing of fludarabine and melphalan with total body irradiation totaling 4 Gray in two fractions. Shortly after the transplant, she developed chest pain in the sternum area described as sharp, non-radiating, and exacerbated with deep breath or position changes. In addition, she noted shortness of breath with exertion, intermittent nausea, and non-bloody, bilious emesis. She denied any vision changes, jaw pain, or epigastric or abdominal pain. The chest pain persisted overnight and the patient was seen by cardiology the following morning.

**Figure 1:**
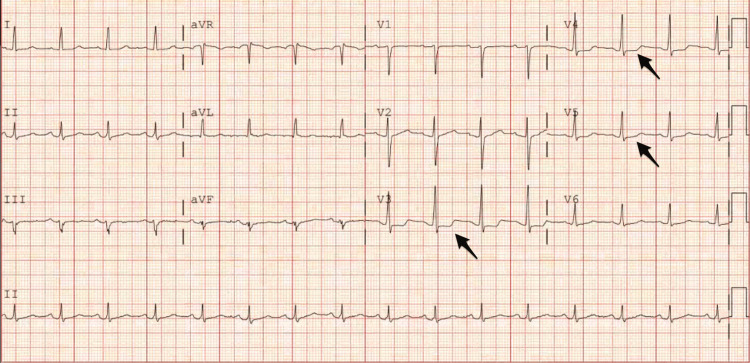
12-lead ECG shows normal sinus rhythm with isolated anterolateral depressions in leads V3-5 (black arrows)

**Figure 2:**
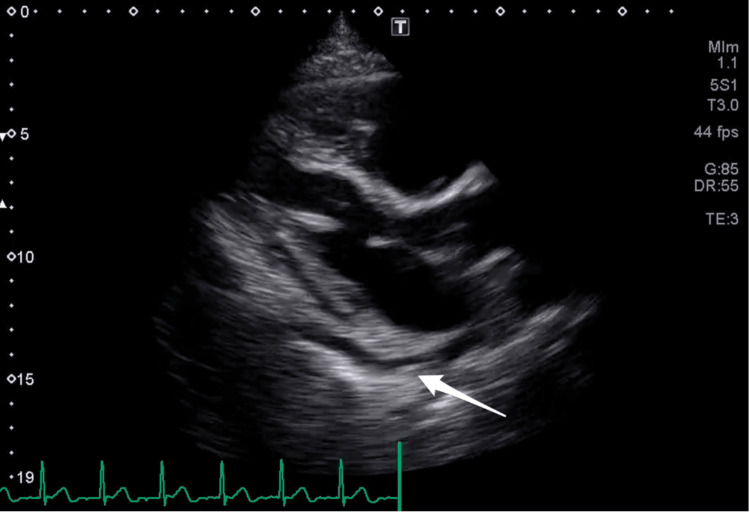
Small pericardial effusion (white arrow) visualized in the parasternal long-axis view of TTE TTE: transthoracic echocardiogram

The patient had no prior history of coronary artery disease, radiation therapy, heart failure, hypertension, diabetes, hyperlipidemia, or thyroid disease. Family history was negative for prior cardiac conditions. Social history was significant for a 13-pack-year smoking history quit 20 years ago. Vital signs showed the patient was normotensive and afebrile with a regular heart rate. On physical exam, heart sounds were normal with no appreciable murmur. Laboratory values revealed a peak troponin of 0.21 ng/mL (0-0.04 ng/mL). Significant anemia was not present and therefore hemolytic markers were not obtained. Electrocardiogram showed dynamic ST depressions in precordial leads V3-5 without evidence of reciprocal changes (Figure 1). The initial transthoracic echocardiography (TTE) showed a small pericardial effusion with preserved ejection fraction and no echocardiographic signs of tamponade (Figure 2, Video 1).

**Video 1:** Parasternal short-axis view of TTE showing normal ejection fraction with no wall motion abnormalities TTE: transthoracic echocardiogram

Post-transplant pericarditis was suspected to be the leading diagnosis. Myocarditis was suggested to be less likely due to the absence of wall motion abnormalities on echocardiography. She was not given non-steroidal anti-inflammatory drugs or colchicine; however, she was promptly started on an intravenous methylprednisolone course at 2 mg/kg daily with a taper regimen over the following two weeks. Her chest discomfort immediately improved and resolved within three days. A transesophageal echocardiogram (TEE) was obtained and revealed normal left ventricular wall motion and contractility with resolution of the pericardial effusion. She was also given metoprolol 12.5 mg orally twice a day for suspected myocardial ischemia, with plans to evaluate for coronary artery disease in the outpatient setting. The patient was unfortunately lost to follow-up.

## Discussion

Cardio-oncology is an emerging field dedicated to describing cancer therapy-related cardiac dysfunction and has thoroughly outlined intermediate- and long-term cardiac risks associated with cancer therapy [8]. Despite the increased awareness, acute cardiac events are rarely reported in the current literature, yet are known to pose a mortality risk [10-12]. In regards to acute pericarditis related to HSCTs, the current literature is limited to two retrospective studies involving a handful of cases [10,13]. Furthermore, there is no consensus surrounding the incidence of acute pericarditis in HSCT recipients. Between the two aforementioned studies, reported incidence rates ranged from 0.9% and 3.2% [10,13]. The limited data available contributes to the obscurity of this potentially life-threatening condition.

Additionally, the pathophysiology of acute pericarditis in the setting of HSCTs remains unclear; however, it can include conditioning-related cardiotoxicity, infectious causes, or complications of graft-versus-host disease. Further, the literature review suggests that polyserositis can be an early complication of endothelial origin, related to complications occurring within the first 100 days of transfusion [14]. This association, however, is specifically described as a complication of microangiopathic hemolytic anemia, the prevalence of which is unclear. Further investigation is warranted to determine whether polyserositis can occur in the absence of anemia, as was the case in this patient. While the etiology of the current patient's presentation is unknown, the temporal association with transfusion suggests a possible infusion-related reaction causing pericardial inflammation.

Acute pericarditis is an uncommon complication of stem cell transplantation that can be fatal if left untreated, prompting urgent management and emphasizing the importance of early recognition and intervention. Within 24 hours of symptom onset, the patient was successfully treated with intravenous high-dose steroids. Although non-steroidal anti-inflammatory drugs and colchicine are often first-line therapies [15], aggressive intervention was preferred in this case due to the potential risk of an atypical presentation of acute graft-versus-host disease.

## Conclusions

This case is unique in the temporal association of pericarditis shortly following the infusion and unusual presentation with non-specific EKG findings. The acuity of acute pericarditis in the early post-transplant phase highlights the importance of cardiac stratification in patients with active malignancy undergoing treatment. It would thus be reasonable to maintain a high clinical suspicion for cardiac events with close monitoring of individuals who have a history of coronary artery disease, smoking, or pericarditis in the pre-engraftment phase of transplantation.
